# Single, very low rituximab doses in healthy volunteers - a pilot and a randomized trial: implications for dosing and biosimilarity testing

**DOI:** 10.1038/s41598-017-17934-6

**Published:** 2018-01-09

**Authors:** Christian Schoergenhofer, Michael Schwameis, Christa Firbas, Johann Bartko, Ulla Derhaschnig, Robert M Mader, Raute Sunder Plaßmann, Petra Jilma-Stohlawetz, Kalpna Desai, Priya Misra, Ulrich Jäger, Bernd Jilma

**Affiliations:** 1 Department of Clinical Pharmacology, Medical University of Vienna, Vienna, 1090 Austria; 2 Department of Medicine I, Division of Hematology and Comprehensive Cancer Center of the Medical University of Vienna, Vienna, 1090 Austria; 3 Department of Laboratory Medicine, Medical University of Vienna, Vienna, 1090 Austria; 4 Apobiologix, Apotex Inc, Toronto, ON M9L 2Z7 Canada

## Abstract

There are no  dose-finding trials available for rituximab that could guide dosing in non-malignant diseases. We hypothesized that currently used doses (≥375 mg/m^2^) exceed several hundred-fold the half-maximal effective dose, which is most sensitive for detecting putative differences between biosimilars and important for dose finding. In an open label, exploratory trial healthy volunteers received single infusions of rituximab at doses of 0.1, 0.3 or 1.0 mg/m^2^. Subsequently, in a double-blind, randomized trial healthy volunteers received single infusions of two rituximab products at doses of 0.1 and 0.3 mg/m^2^. In the exploratory trial rituximab transiently depleted CD20+ cells by a mean 68% (range: 57–95%), 74% (55–82%) and 97% (94–100%) immediately after the infusion of 0.1 (n = 4), 0.3 (n = 4) and 1 mg/m^2^ (n = 8), respectively. In the randomized trial CD20+ cells decreased by a mean 48% (25–84%) − 55% (26–85%) and 81 (67–89%) – 87% (77–96%) after infusion of 0.1 mg/m^2^ (n = 12) or 0.3 mg/m^2^ (n = 8 proposed biosimilar, n = 4 reference product) of the proposed biosimilar or the reference product, respectively. It is important to understand that in healthy volunteers <1% of the authorized rituximab doses depletes almost all circulating B lymphocytes. Thus, for non-malignant diseases alternative, more cost-effective dosing regimens seem plausible, but require clinical testing. (EudraCT-No. 2010–023781–45; EudraCT-No. 2013–001077–24).

## Introduction

Rituximab is a highly specific, chimeric, monoclonal CD20 antibody^1^. The CD20 antigen is expressed on normal B-cells, pre-B cells, as well as on B-cells in chronic lymphocytic leukemia, in >90% of B-cells in Non-Hodgkin lymphomas and on >50% of B-cells in acute lymphocytic leukemia^2^.

Rituximab is approved for use in hematological malignancies, with a dose regimen of 375 mg/m^2^ every four weeks^3^, and in rheumatoid arthritis, with a dose regimen of 2 × 1000 mg^4,5^. However, rituximab is also frequently used “off-label” in the treatment of antibody-dependent auto-immunological diseases including but not limited to autoimmune haemolytic anemia^6,7^, idiopathic thrombocytopenic purpura^8^, thrombotic thrombocytopenic purpura^9^, neuromyelitis optica and multiple sclerosis^10,11^, pemphigoid diseases^12^, and possibly nephrotic syndrome^13^. In some of these off-label indications alternative dosing schedules, i.e. 4*100 mg rituximab/week^7,14^, are used. *In vitro* data suggested that the half maximal effective concentration (EC50) of rituximab in humans is ≤1 µg/ml^15^. Thus, all regulatory approved doses of rituximab, exceed that plasma level at least 200–300-fold^1^.

Noteworthy, a polymorphism of FcγRllla (CD16) influences the efficacy of rituximab^16^. Whether this polymorphism affects clinical outcomes is still unclear^16^.

Various biosimilar products of rituximab are currently under development. The European Medicines Agency and the US Food and Drug Administration have published guidance on the development of biosimilars recommending a stepwise approach, for the totality of evidence, addressing structural aspects of the product, functional assays, animal studies, pharmacokinetic and pharmacodynamic properties in humans, immunogenicity analysis and possibly clinical efficacy and safety trials^17,18^. Evaluation of effects should be performed in the steep part of the dose-response curve, which is typically close to the EC50^17,18^. However, the optimal dose to compare rituximab products is currently unknown. As B-cell depletion is the only effect of rituximab, the aim of this trial was to investigate a dose at which comparing the effects of biosimilar rituximab products is most sensitive. Based on the low *in vitro* EC50^15^ we hypothesized that tiny fractions of authorized rituximab doses would be sufficient to achieve that in humans. This was first investigated in a pilot trial. The second trial compared the B lymphocyte depletion, immunogenicity profile and safety of a proposed biosimilar rituximab product to MabThera®, (authorized rituximab, Roche, Basel, Switzerland) in an exploratory, randomized, double-blind, active controlled trial in healthy volunteers.

## Results

Recruitment of healthy volunteers for both trials was between March 29^th^ 2011 and May 23^rd^ 2013. The trial was ended after the last follow-up visit on October 29^th^ 2013. Demographic data and subject disposition are presented in the Supplement (Tables S1 and S2).

### Pilot Trial

**Figure 1:**
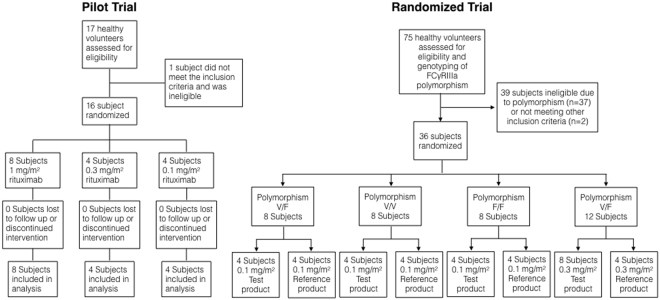
Flowchart of the pilot and the randomized, double blind trial with stratification for the FCγRIIIa-158V/F polymorphism V/F, V/V and F/F.

A total of 16 Caucasian subjects (ten males and six females) were enrolled in the first trial with a mean age of 32 (range 20–49) years, a mean height of 176 (163–191) cm and a mean weight of 74 (55–98) kg (flowchart Fig. 1).

The pharmacokinetics of rituximab at 1 mg/m^2^ is shown in Table 1. Figure S1 shows the plasma concentration curve (Supplement). Due to limitations with the analytical sensitivity of the ELISA method, it was not possible to determine the pharmacokinetics of rituximab at doses 0.3 and 0.1 mg/m^2^. Additionally, even at a dose of 1 mg/m^2^ the elimination half-life could not be reliably calculated due to the limits of the analytical assay at this low-dose regimen. Therefore, the observation period was truncated after 24 hours.

**Table 1:** Pharmacokinetics of rituximab at 1 mg/m^2^ (N = 8).

**Parameters**	**N**	**Mean**	**Minimum**	**Maximum**	**CV%**
AUC_0–24_ [ng*h/ml]	8	1599	1371	1908	12.7
AUC_0–24_/ D [m^2^*h/ml]	8	0.0016	0.0014	0.0019	12.7
C_max_ [ng/ml]	8	289	169	431	26.9
C_max_ / D [m^2^/ml]	8	0.00029	0.00017	0.00043	26.9
T_1/2_ [h]	8	36	18	71	53
CL [ml/m^2^*h]	8	634	524	729	12

PK parameters after infusion of 1 mg/m^2^ rituximab in 8 healthy volunteers (PP population). AUC_0–24_ = Area under the curve of plasma rituximab concentrations for the first 24 hours after infusion; AUC_0–24_/D = Area under the curve of plasma rituximab concentrations for the first 24 hours per Dose; C_max_ = maximum concentration; C_max_/D = maximum concentration per Dose; T_1/2_ = terminal elimination half-life; CL = Clearance;

*multiplication sign.

**Figure 2:**
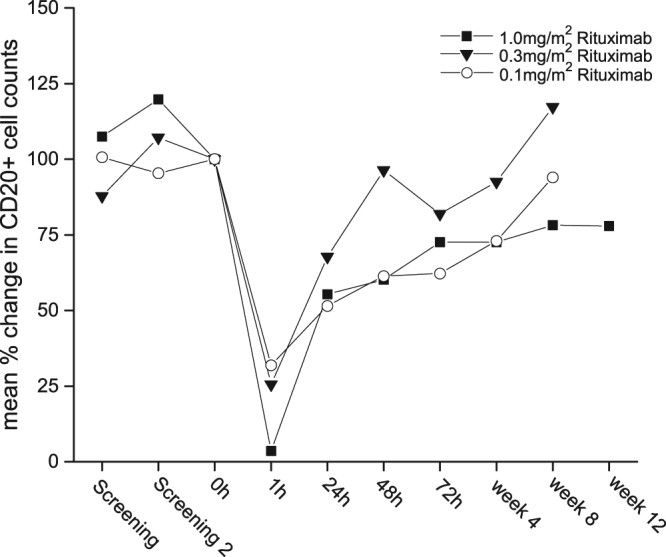
Mean relative changes in CD20+ cell counts after infusion of rituximab at 1 mg/m^2^ (n = 8), 0.3 mg/m^2^ (n = 4) or 0.1 mg/m^2^ (n = 4). Screening = first investigation, Screening 2: one week before rituximab infusion, 0 h = before infusion of rituximab, 1 h = end of infusion, 24 h = 24 h after start of infusion, 48 h = 48 h after start of infusion, 72 h = 72 h after start of infusion, week 4 = 4 weeks after start of infusion, week 8 = 8 weeks after start of infusion, week 12 = 12 weeks after start of infusion.

Rituximab almost immediately depleted CD19/20+ cells from the systemic circulation at the end of infusion (Fig. 2). Mean CD20+ cell counts decreased by approximately 97% (95% CI: 95–99%, range: 94–100%) from the baseline after infusion of 1 mg/m^2^, 74% (95% CI: 59–84%, range: 55–82%) and 68% (95% CI: 24–100%, range: 57–95%) after infusion of 0.3 mg/m^2^ or 0.1 mg/m^2^, respectively. This drop was transient and showed a quick but incomplete recovery after 24 hours. Four weeks after infusion of rituximab CD20+ cells returned to approximately 60% of baseline levels in the 1 mg/m^2^ dose group. Remarkably, full recovery took up to nine months in this dose group, whereas CD20+ counts fully recovered in the lower dose groups four weeks after infusion.

### Second Trial

Thirty-six Caucasian subjects were included (22 males and 14 females) in the second trial with a mean age of 31 (20–49) years, a mean height of 175 (157–194) cm and a mean weight of 70 (48–107) kg.

As the observed confidence intervals (CI) were very tight for the 1 mg/m^2^ dose, the second trial included two dose cohorts at the dose level 0.1 mg/m^2^ (24 subjects) and 0.3 mg/m^2^ (12 subjects) (Fig. 1).

The coefficient of variation of CD20+ cells at baseline ranged from approximately 22–61% in the different groups (Table S4).

**Figure 3:**
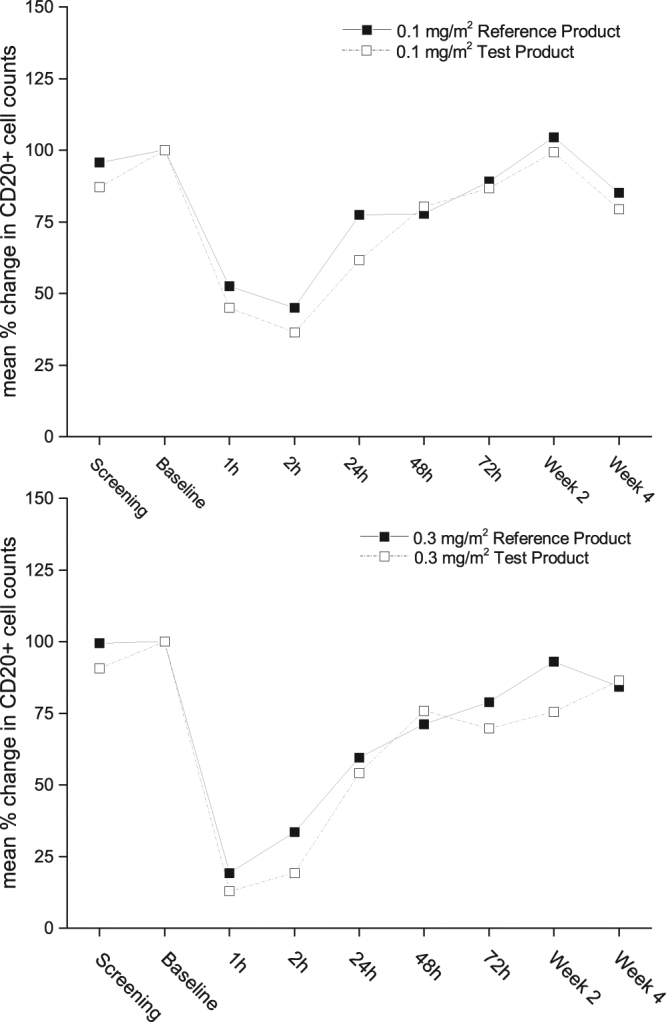
Mean % changes from baseline in CD20+ cell counts after infusion of 0.3 mg/m^2^ (n = 4 for the reference product, n = 8 for the test product) and 0.1 mg/m^2^ (n = 12, per group) of the rituximab test or reference product are presented. Time-points: Screening visit, Baseline: before infusion, 1 h: End of infusion or 1 h after start of infusion, 2 h: 2 h after start of infusion, week 2 after infusion, week 4 after infusion.

At a dose of 0.1 mg/m^2^ rituximab reduced the CD20+ cell count by a mean of 48% (CI 95% 32–58%, range 25–84%) and 55% (CI 95% 45–66%, range 26–85%) after infusion of the proposed biosimilar or the reference product, respectively (Fig. 3). At a dose of 0.3 mg/m^2^ rituximab decreased CD20+ cell counts by a mean of 81% (CI 95% 73%-87%, range 67–89%) and 87% (CI 95% 74–100%, range 77–96%) after infusion of the proposed biosimilar or the reference product, respectively (Fig. 3). The overlapping confidence intervals indicate no statistical differences.

The coefficient of variation of the relative decrease in CD20+ cells after infusion of 0.1 mg/m^2^ was 35% and approximately 10% after infusion of 0.3 mg/m^2^ rituximab for pooled data of both products.

Infusion of rituximab at 0.1 mg/m^2^ had the numerically greatest effect in subjects with a V/V genotype and reduced CD20+ cells by approximately 62%, compared with 48% of the V/F genotype and 42% of the F/F genotype (Table S3). These differences were not statistically significant.

In most subjects, rituximab serum concentrations were not quantifiable beyond two hours in the lowest dose group and seven hours in the 0.3 mg/m^2^ dose group. Half-lives (T_1/2_), and consequently V_d_ could not be calculated due to low drug levels.

The detected C_max_ varied moderately with CV <35% for both rituximab products at 0.1 mg/m^2^ and <40% at 0.3 mg/m^2^. Statistical comparison was only performed at doses of 0.1 mg/m^2^ and no significant differences were found between the test and the reference product regarding the parameters AUC_0–24_ (p = 0.7), AUC_0-inf_ (p = 0.12) and C_max_ (p = 0.2). At the higher dose group, statistical comparison was not performed due to the low number of subjects. Thus, the comparative data is presented and interpreted for information purposes only.

### Immunogenicity

Human anti-chimeric antibodies (HACA) were positive in three subjects before infusion of rituximab. After treatment with low doses of rituximab, 26 of 36 subjects were positive for HACA, of whom 14 had been treated with the proposed biosimilar product and 12 with the reference product.

Neutralizing antibodies were only tested in patients who were positive for HACA. At the final safety visit, neutralizing anti-drug antibodies (NADA) were detected in nine of 36 subjects, of which five received the proposed biosimilar product and four the reference product.

### Safety

In both trials, a total of 106 non-serious, non-severe AEs were documented (Supplementary Tables S6 and S7). Among them, 56 AEs were graded as moderate and 50 as mild with 33 suspected to be related to the trial drugs. All but two AE resolved until the final safety visit. All occurring AEs have been described earlier to be associated with rituximab exposure.

With regard to laboratory parameters similar changes in both trials were detected. A significant but transient and dose dependent increase in C-reactive protein was detected after rituximab exposure (Table S8). Hemoglobin, white blood cells and lymphocytes dropped below baseline immediately after infusion of rituximab. These changes were minor, graded as not clinically relevant and resolved until the final safety visit. In both trials, a short-termed decrease of C3, C4 and CH50 during and shortly after infusion was noticed, which was compensated within one day. This recovery was independent of dose, polymorphism or rituximab product for the complement factors under investigation.

## Discussion

For the first time, we investigated the dose-response relationship of rituximab at very low doses, which is important for the most cost effective use of rituximab for off-label treatment in non-malignant diseases. The half-maximal effective dose for depletion of circulating CD20 + B-cells was 0.1 mg/m^2^, which is only a tiny fraction (1:3750–5000) of the authorized doses.

We chose a starting dose of 1 mg/m^2^ to identify the half maximal effective dose *in vivo*, because the half maximal effective concentration is ≤1 µg/ml *in vitro* ^15^. As mentioned above, this dose is of particular interest, because it is the most sensitive dose to detect differences in the effects between biosimilar rituximab products and may guide dose-finding. Infusion of 1 mg/m^2^ rituximab already depleted 97% of all B-cells in healthy volunteers. Thus, instead of increasing the dose of rituximab as originally planned, the dose was reduced to 0.3 mg and 0.1 mg/m^2^. These doses still lowered CD20+ cell counts by 75% and 66% of baseline values in the pilot trial. Our results suggest that comparison of the effects of rituximab in the steep part of the dose-response curve is no longer possible when doses reach 1 mg/m^2^.

**Figure 4:**
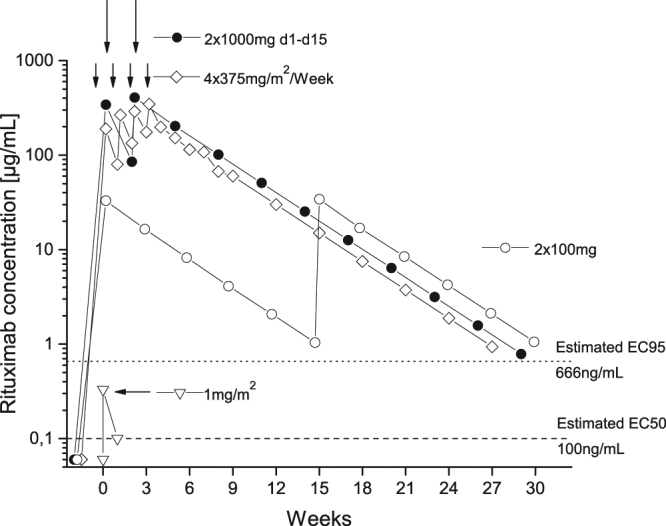
A pharmacokinetic model: plasma rituximab concentrations with different dosing regimens. Arrows indicate infusions of rituximab. Data were adapted from Cohen *et al*.^30^ 2 × 1000 mg (day 1 and day 15), Iacona *et al*.^3^ 4 × 375 mg/m^2^/week and extrapolated from data of this trial (1 mg/m^2^ and 2 × 100 mg (smallest vial size)).

The demonstrated high potency of rituximab at very low doses may have important implications for rituximab dosing in non-malignant diseases. The ≥95% depletion of circulating B lymphocytes after infusion of 1 mg/m^2^ rituximab, although of a transient nature, makes it conceivable that lower doses than the currently authorized (≥375 mg/m^2^) may suffice to deplete all B lymphocytes from the circulation and therefore may be equally effective. However, this hypothesis needs to be tested in further clinical trials. This is especially interesting for low-income countries struggling to meet the financial burden of treatment with biologicals^19^. We extrapolated these data and hypothesize that 100 mg rituximab (lowest strength available) may suffice to fully suppress CD20+ cells for three months (refer to the pharmacokinetic model and Fig. 4). Assuming a price of approximately 1.75 Great Britain Pounds (GBP) per mg rituximab^20^, two doses of 1000 mg in rheumatoid arthritis could result in drug costs of approximately 3500 GBP. Two doses of 100 mg (i.e. the smallest vial size) every three months would add up to 350 GBP, a cost reduction of 90%. However, these are only extrapolations from healthy volunteers that require validation in clinical trials involving patients. The ORBIT study reported that annual costs decreased by ~20% when rituximab was used instead of a TNF-α inhibitor in patients with rheumatoid arthritis^21^. Alternative dosing schedules could decrease the costs further.

No differences in immunogenicity were found between the two rituximab products regarding development of anti-drug antibodies. However, the incidence rates of these antibodies were higher in our trial than reported previously. The rate of human anti-chimeric antibodies after infusion of rituximab was 33% in patients with systemic lupus erythematodes^22^, 4.3% (5 out of 117) in patients with rheumatoid arthritis^23^ and less than 1% (1 out of 166) in patients with indolent lymphoma^24^. The frequency of anti-drug antibodies in multiple sclerosis patients was approximately 30%^25^. Noteworthy, such patients often receive other immunosuppressive drugs such as methotrexate, glucocorticoids and chemotherapy. These findings should not impose any risks on patients in clinical trials of low-dose rituximab, because in contrast to our trial, in these patients a complete and continuous depletion of all B lymphocytes is pursued. Thus, the initial target dose of the first infusion should ideally remove all CD20 + B lymphocytes. In such case, we assume that anti-drug antibody generation should not differ from other patient populations despite of the low doses. However, based on our findings we recommend testing for anti-drug antibodies in all trials investigating lower doses of rituximab.

For biosimilarity testing, the experimental setup of a submaximal depletion of B lymphocytes may be especially suitable to detect and to compare immunogenicity, because high rituximab doses completely deplete B-cells, which may suppress the humoral immune response to the drug itself and possibly bias the immunogenicity results.

A multitude of impediments hinders the conduct of pivotal clinical trials for the introduction of new drugs to the market. These obstacles include increasing financial and regulatory burdens by the need of multinational multicenter trials, unwillingness of many patients to enroll in clinical trials, as well as diminishing incentives for investigators in health systems focusing on efficiency and profitability^26^. In some instances such as testing of biosimilarity, alternative approaches to these extensive trials such as extrapolation may be a reasonable approach^27^. In certain cases, the results of human, pharmacological studies, as well as clinical immunogenicity testing may suffice for approval of biosimilarity^18,28,29^. Increasing the validity of the pharmacodynamic biosimilar exercise may help to decrease the burden for the phase III comparability exercise and therefore the costs of the development program.

Some limitations need to be considered. First of all, the number of participants was small and this exploratory trial was not powered to formally assess biosimilarity between the two rituximab products using established non-inferiority margins.

Some technical limitations related to the sensitivity of the pharmacokinetic assay occurred and rituximab was detectable only for a short period of time at low doses. As a result, it was not possible to accurately assess pharmacokinetic properties of rituximab at low doses. There was a clear pharmacokinetic signal for the rituximab dose of 1 mg/m^2^ during the first 24 hours. Thus, the calculated mean T_1/2_ of 36 hours is markedly shorter than the mean rituximab T_1/2_ of approximately 20 days after 375 mg/m^2^ or 1000 mg^3,30^. For the same reasons pharmacokinetic analysis at lower doses (≤0.3 mg/m^2^) was technically not feasible.

Another limitation of our trial was that the effects of neutralizing anti-drug antibodies on the efficacy after repeated exposure to low dosed rituximab could not be investigated as rituximab was only infused once in healthy volunteers for safety reasons.

While in our study CD19 and CD20 counts behaved similar in almost all subjects, this need not be the case if higher doses of rituximab are used or other detection antibodies are used in the laboratory. Ideally clinical laboratories will have to check for the *in vitro* interference of rituximab with their antibodies used to detect CD20+ cells up to maximum plasma concentrations, i.e. 500 µg/mL.

These data are derived from a trial in healthy volunteers. There are no clinical efficacy data and proposed new dosing regimens are based on extrapolations and require well designed clinical trials.

## Conclusion

In conclusion, even very low doses of rituximab ≤1 mg/m^2^ effectively depleted CD20+ cells in healthy volunteers. Based on these data, alternative, more cost-effective dosing regimens seem plausible for the use of rituximab in non-malignant diseases. However, efficacy of such regimens needs to be tested in further clinical trials involving patients and pharmaco-economic evaluations. Considering the world-wide use of rituximab, such trials are desperately needed and may be particularly relevant for low-income countries struggling to meet the financial burden of therapy with biologics.

## Methods

### Trial Design

Two clinical trials were conducted (Fig. 1). The first trial was a single center, open label, dose-escalation trial evaluating the optimal dose in healthy volunteers. We explored the half-maximal effective dose (ED50) by infusion of 1 mg/m^2^, 0.3 mg/m^2^ and 0.1 mg/m^2^ rituximab. The second trial was a single center, stratified (FCγRIIIa polymorphism), randomized, double blind, active controlled trial, where the effects, the safety and the immunogenicity of two rituximab products were compared, the reference product (MabThera®, Roche, Basel, Switzerland) and the proposed biosimilar product (Apotex Inc., Toronto, Canada) at the previously identified optimal doses. The national competent authority of Austria (AGES, Austrian Agency for Food and Drug Safety) and the Independent Ethics Committee of the Medical University of Vienna approved both trials, which were conducted in compliance with Good Clinical Practice and the Declaration of Helsinki. The trials were registered in a public database with the identifiers EudraCT No. 2010-023781-45 on November 5^th^ 2010 and EudraCT No. 2013-001077-24 on March 14^th^, 2013. Detailed information on the study design is presented in the supplement. The trial protocols are available in the supplement. Individual data are available upon request.

### Participants

The Department of Clinical Pharmacology at the Medical University of Vienna, Austria, enrolled healthy male and female volunteers aged 18–55 years, 16 for the first trial and 36 for the second trial. Oral and written informed consent was obtained before any study-related activity began.

### Randomization and Masking

An independent statistician created a randomization list applying block randomization. A study nurse, who enrolled all participants, assigned subject numbers. Individual randomization codes and treatment allocation were concealed until drug dilution immediately before administration. To maintain blindness of participants and investigators, a pharmacist, not otherwise involved in the trial, prepared study medication in syringes, which were all labeled with “rituximab” and indistinguishable from each other.

### Procedures

All healthy volunteers reported to the ward after an overnight fast, where they were confined until the afternoon of the same day. Thirty minutes after pre-medication with acetaminophen (500 mg p.o., Paracetamol, Genericon Pharma GmbH, Graz, Austria) and levocetirizine (5 mg p.o., Genericon Pharma GmbH, Graz, Austria), rituximab was administered. The reference or the proposed biosimilar product was infused over a period of 1 hour. In the first trial only the reference product (MabThera®) was infused. Vital signs were monitored closely.

Blood sampling was performed from an antecubital intravenous catheter (opposite of the infusion arm). In both trials CD19/20+ cell counts and rituximab concentrations were assessed at predefined time-points (Supplement for details).

### Outcomes

In the pilot trial the area under the plasma concentration curve (AUC_0–24_) was the primary endpoint, with other pharmacokinetic parameters (Clearence Cl, maximum concentration C_max_, elimination half-life T_1/2_, AUC per Dose AUC/D) and CD19/20+ cell counts comprising secondary endpoints. In the second trial the primary endpoint was CD19/20+ cell counts, while secondary endpoints comprised pharmacokinetics (for details see supplement).

### Statistical Analysis

No formal sample size calculation was performed, because of the expected high magnitude of pharmacodynamic effects. For the pilot trial we initially planned a staggered approach (n = 3 healthy volunteers per group, with a maximum of 8 healthy volunteers in the highest dose group). The second (n = 4) and third dose group (n = 4) eventually received lower doses, because the first dose (1 mg/m^2^) provided already stronger than expected efficacy.

The sample size calculation for the second trial was chosen to obtain a reliable estimate for the variability of the reduction in CD20+ cells. As the variability of absolute cell counts was fairly high, relative changes were calculated.

The confirmatory randomized trial was planned to obtain a better estimate of the inter-subject variability of the response to rituximab with emphasis on the test product. For this purpose at least 18 subjects receiving the test product and up to 16 subjects receiving the reference product MabThera® were included (12 subjects in total for 0.3 mg/m^2^ rituximab and up to 24 volunteers for 0.1 mg/m^2^ rituximab).

Results of the pilot trial were only interpreted using descriptive statistics.

For the second trial for inferential analyses treatment groups of 0.1 mg/m^2^ rituximab dose were combined across all genotypes. The values of the main pharmacodynamic (minimum of CD20+ cells) and pharmacokinetic parameters (AUC_0–24_, AUC_0-inf_, C_max,_ Cl) were compared using ANOVA with the fixed factor treatment and a significance level of α = 0.05 with the parameters AUC and C_max_ to be transformed prior to analysis using a logarithmic transformation (exponential).

All statistical analyses were performed using SAS^®^ Version 9.2.

### Clinical Trial Registry Information

The trials have been registered at EudraCT database with the identifiers EudraCT-No. 2010-023781-45 on November 5th 2010 and EudraCT-No. 2013-001077-24 on March 14th 2013.

## Electronic supplementary material

Extended Materials and Methods and Results
