# Supplementary material for: Single, very low rituximab doses in healthy volunteers - a pilot and a randomized trial: implications for dosing and biosimilarity testing
Source: Sci Rep. 2018 Jan 9;8:124. doi: 10.1038/s41598-017-17934-6 (PMC5760686; doi:10.1038/s41598-017-17934-6)
Supplement: Supplementary file 1 — Extended Materials and Methods and Results [file 41598_2017_17934_MOESM1_ESM.pdf]

Single, very low rituximab doses in healthy volunteers - a pilot and a randomized trial: implications for dosing and biosimilarity testing

Christian Schoergenhofer, MD[a], Michael Schwameis, MD[a], Christa Firbas, MD[a], Johann Bartko, MD[a], Ulla Derhaschnig, MD[a], Robert M. Mader, Phd[b], Raute Sunder-Plaßmann, MD[c], Petra Jilma-Stohlawetz, MD[c], Kalpna Desai, PhD[d], Priya Misra, PhD[d], Ulrich Jäger, MD[b] and Bernd Jilma, MD[a]

[a] Department of Clinical Pharmacology, Medical University of Vienna, 1090, Austria

[b] Department of Medicine I, Division of Hematology and Comprehensive Cancer Center of the Medical University of Vienna, 1090, Austria

[c] Department of Laboratory Medicine, Medical University of Vienna, 1090, Austria

[d] Apobiologix, Apotex Inc.Toronto, ON M9L 2Z7, Canada

Supplements

Study design

To ascertain the pharmacological effects of rituximab in a healthy subject population, two trials were conducted. The first trial was a single center, non-blinded, dose-escalation study evaluating the optimal dose in healthy adult male and female volunteer subjects. To determine the optimal dose a stepwise dose-escalation trial starting at 1 mg/m^2 with maximal doses of 16 mg/m^2 was planned. Preclinical *in vitro* data indicated a half-maximal effective concentration (EC50) for rituximab lower than 1 μg/ml.[11] Thus, the dose escalation started at a dose of 1 mg/m^2 to provide roughly a maximal concentration of 500 ng/ml, due to the significantly higher depletion of B-cells at the lowest dose level of rituximab (first eight subjects planned and dosed), the rituximab dose was not escalated further, but was reduced to 0.3 mg/m^2 rituximab (four subjects) and 0.1 mg/m^2 rituximab (four subjects). Subjects received single doses: 1 mg/m^2 or 0.3 mg/m^2 or 0.1 mg/m^2 of MabThera®. The total study duration per subject was approximately 16 weeks. The trial period was from March 29th 2011 to January 9th 2013. In consideration of the results from the dose escalation trial the second comparative sub-therapeutic pharmacokinetic (PK) and pharmacodynamic (PD) trial was conducted in healthy adult male and female subjects at dose levels of 0.3 mg/m^2 and 0.1 mg/m^2. This was a stratified, randomized, subject and observer- blinded, active controlled, parallel group pilot study. The recruitment period lasted from May 23rd 2013 until October 29th 2013. The total study duration was approximately 16 weeks. In both trials rituximab was administered over an hour as intravenous administration. The first cohort of subjects was stratified into three groups according to their FCγRIIIa-158V/F polymorphism and was randomized 1:1 to receive either the proposed rituximab biosimilar product or the reference product MabThera® at a dose of 0.1 mg/m^2. As soon as eight subjects with the genotype V/F had received 0.1 mg/m^2 rituximab, the dose was escalated and the next 12 subjects with genotype V/F were randomized 2:1 (n=8 for proposed rituximab biosimilar product and n=4 for MabThera®) to

receive a dose of 0.3 mg/m^2 rituximab. Randomization to the 0.1 mg/m^2 dose group was continued for subjects with the genotypes V/V or F/F until eight subjects of each genotype had been treated in that dose cohort.

In- and Exclusion Criteria

In both trials volunteers also had to meet the following inclusion criteria: normal findings in medical history, physical examination and laboratory values and female subjects either childbearing potential terminated by surgery or one year post- menopausal, or a negative urine pregnancy test during screening and the willingness not to become pregnant during the entire study period by practicing reliable methods of contraception as described in the protocol. Exclusion criteria included treatment with an investigational drug within one month prior to this trial; recent infection (within one week); relevant history of renal, hepatic, gastrointestinal, cardiovascular, respiratory, skin, hematological, endocrine, inflammatory or neurological diseases; ascertained or presumed hypersensitivity to the active principle and/or formulations' ingredients; history of anaphylaxis to drugs or major allergic reactions in general. Additional exclusion criteria for the second trial included a pre-screening visit, which was conducted to test for the FCγRIIIa-158V/F polymorphism. If subjects had a genotype (V/V, V/F, F/F), which was required for randomization, a screening visit was performed up to four weeks before infusion of the drug.

Blood Sampling

In both trials, blood sample were collected in polypropylene tubes containing EDTA (1 mg EDTA/mL of blood; Vacuette®) and kept at room temperature (20 °C ± 4 °C). For rituximab, blood samples were centrifuged (2000xg at 4 °C for 15 minutes). Collected plasma (first trial) and serum (second trial) were divided into two aliquots of at least 0.5 mL each and stored at -80 °C until analysis. Plasma or serum rituximab levels were quantified by a qualified enzyme immunoassay (ELISA); CD19 and CD20 positive lymphocytes were determined by flow-cytometric analysis (FACS) at the institution's central laboratory to exclude competition of

rituximab with the CD20 antibody used for the flow cytometric assay. CD19/20+ cell counts were repeated one week before infusion of the trial drug. CD20+ cells results are reported as CD19 and CD20 results were similar

PK parameters were calculated from original data using commercially available software (Kinetica™, InnaPhase Corporation, Philadelphia, USA).

Anti-rituximab antibodies were detected by electrochemiluminescence-based immunoassay method. Neutralizing anti-drug antibodies were detected by a complement dependent cytotoxicity assay

Blood Sampling Schedule:

In the first trial, at all dose levels, the pharmacodynamic effects of rituximab, CD19/20+ cell count was assessed at pre-dose, and at the end of infusion, Day 1, Day 2, Day 3, Week 4, Week 8, and at Week 12*, Month 6* and Month 9* (*optional visits dependent on B cell recovery) after administration. In the second trial, CD20 cell counts were assessed at pre-dose, at the end of infusion and at 1h±5mins, at 24h, once weekly from week 1 to 4, thereafter on a monthly basis up to week 12. As CD20+ cell counts did not fully return to baseline levels in all subjects, additional blood samples were taken three and six months after the last visit. The last visit was three months after infusion of rituximab, the full observation period was therefore nine months, unless the CD20+ cell levels returned to the normal range earlier (defined as baseline±10%). In both trials, rituximab concentrations were measured at pre-dose, at the end of infusion, and 15mins, 1h±5mins, 2h±5mins, 4h±5mins, 6h±5mins, 24h±1h, 48h±1h, 72h±1h and once weekly up to week 8 (week 4 for the second trial) thereafter.

CD19/20+ cell counts

Gating strategy: Normally 20000 events are acquired, but a minimum of 2000 lymphocytes are gated. If the B cell count is low, 50000 lymphocytes are acquired to permit sufficient confidence that the CD20+ B-cell count is less than 0.1%. Both, CD19+ and CD20+ cells were counted.

Rituximab enzyme-linked immunosorbent assay

Rituximab concentrations were determined using enzyme-linked immunoassay (developed at Vela Laboratories, Vienna, Austria for the first trial and at PPD Richmond, VA, USA for the second trial). Anti-idiotype rituximab antibody (MB2A4) was coated to the ELISA plate. After blocking, human plasma samples (diluted 1:50 or 1:100) were added to the wells to allow rituximab binding to the coated antibody. The detection antibody, mouse anti-human IgG Fc-HRP, was added. Detection was based on horseradish peroxidase (HRP) conversion of the substrate 2,2'-azino-bis-(3-ethylbenzthiazoline-6-sulphonacid) diammonium salt (ABTS) into green chromogenic product in the presence of H_2O_2. Absorbance was measured at 405 and 492 nm wavelength with an ELISA reader; OD values at 492 nm were used to subtract the baseline from OD at 405 nm. The results were used to plot a standard curve for rituximab detection (assay range: 0.313 ng/ml to 5 ng/ml of diluted rituximab in plasma) and calculate rituximab concentration in human plasma samples based on this curve.

Presented values were baseline corrected by subtracting baseline noise from all subsequent rituximab concentrations.

FCγRIIIa-158 polymorphism

DNA was purified according to standard procedures. The analysis of the FCγR3A SNP rs396991 was performed by Real-Time PCR and melting curve analysis using the LightSNiP rs396991 assay (TibMolbiol, Berlin, Germany) and the LightCycler FastStart DNA Master Hybridisation Probes Kit (Roche Diagnostics, Penzberg, Germany) on a Light Cycler 2.0 (Roche) according to the manufacturer's suggestions.

Human anti-chimeric Antibody (HACA) detection

We detected anti-rituximab antibodies by electrochemiluminescence-based immunoassay method (Vela Laboratories Vienna, Austria for the first trial and at PPD Richmond, VA, USA for the second trial). Samples were diluted with 300mM acetic acid and incubated with

conjugate/neutralization mixture consisting of biotin-labled Rituximab, sulfo-TAG-labeled rituximab, 1 M Tris pH 9.5 and soluble drug. During incubation the two antigen binding sites of anti-rituximab antibodies are able to form a bridge between the labeled Rituximab antibodies for detection. The sample mixture is then added to a blocked streptavidin-coated surface of the well resulting in the immobilization of the bridged complex. After washing tripropylamine MSD read buffer is added to each well. Using the MSD 6000 plate reader, an electrical current is introduced across the plate-associated electrodes resulting in oxidation-reduction reactions involving ruthenium and tripropylamine leading to a luminescent signal.

Neutralizing anti-drug antibodies (NADA)

NADA were detected by a complement dependent cytotoxicity assay (Vela Laboratories, Vienna, Austria and PPD Richmond, VA, USA). This assay is based on complement system activation by binding of rituximab to CD20 antigen on B-cells. To inactivate the endogenous serum complement activity, samples were heat-inactivated at 56±2°Celsius for approximately 30 minutes. Samples were incubated with rituximab for 2 hours at room temperature. Any neutralizing drug antibodies present in the samples were allowed to form antibody complexes. Next, samples were treated with a 1:2 dilution of normal human serum complement in the presence of WIL2-S cells. After incubation for 2.5 hours at 37±2°Celsius with 5±1%CO2, the number of live metabolically active cells in each well is measured by quantifying the ATP present using the reagent CellTiter-Glo®. Luminescence was measured using the Wallac EnVision. The signal produced is directly proportional to the amount of anti-rituximab neutralizing antibodies.

Safety

Safety measurements included documentation of adverse events, laboratory tests and vital signs and were performed throughout the trial. Immunogenicity assessment was performed prior to dosing and four weeks post-dose in the safety follow up visit. Before inclusion a full physical examination, height and weight measurements, heart rate, systolic/diastolic blood

pressure measurement, oxygen saturation, oral temperature determination and a 12-lead electro-cardiogram were performed. Blood and urine analysis was performed including blood counts, blood chemistry, coagulation parameters, virology, IgM and IgG levels, presence of HACA, complement levels and complement activity, screening for illegal drugs, a urine pregnancy test and urinalysis. Furthermore, all participants received vaccination against diphtheria, tetanus, whooping cough (pertussis) and polio (Repevax®), if the last vaccination was >5 years ago. Additionally, a live virus vaccine for immunization against measles, mumps and rubella (Priorix®) was administered, if subjects had not received this vaccination in childhood. If subjects had no detectable antibody levels at day -4 and day 0, they received full vaccination according to the vaccination schedule (vaccination in week 4, 8 and 6 months after first vaccination).

The final safety follow-up visit was performed 28±3days after infusion. However, if additional post-study visits were necessary, due to persistent low CD19/20+ cell counts, adverse events were recorded if they occurred during this prolonged follow-up period.

Endpoints

In the first trial the primary PK endpoint was the area under the plasma concentration-time curve (AUC_t). Other parameters included AUC/D, peak concentration (C_{max}), the apparent elimination half-life ($T_{1/2}$), and the rate at which the drug is cleared from the body (CL). The concentration-time profile of rituximab was assessed up to four weeks after infusion. Secondary endpoints comprised CD19+ and CD20+ cell counts as the main PD parameter. Safety parameters included safety parameters IgM, IgG levels, complement levels (C3, C4, CH50), differential blood counts, serum chemistry (K, LDH, uric acid), tryptase and HACA. As tertiary endpoints a comparison of systemic safety (adverse events, laboratory parameters, vital signs) was performed

In the second trial the primary endpoints were CD19+ and CD20+ cell counts and pharmacokinetic parameters (AUC, AUC/D, Cl, $C_{max,}$ V_d and $T_{1/2}$) comprised secondary

endpoints. The concentration-time profile of each rituximab product was assessed up to four weeks after infusion.

Tertiary endpoints included systemic safety (adverse events, laboratory parameters, vital signs, electrocardiogram), IgM and IgG levels, complement levels (C3, C4, CH50), differential blood counts, serum chemistry (K, LDH, uric acid, tryptase), HACA (human anti-chimeric antibody) and NADA (neutralizing anti-drug antibody).

Baseline data and subject disposition

First trial

Of 17 screened healthy volunteers, 16 healthy volunteers were eligible for inclusion into the pilot trial. Eight subjects received 1.0 mg/m^2, four subjects received 0.3 mg/m^2 and four subjects received 0.1 mg/m^2 rituximab (Mabthera). Of six female subjects three received 1mg/m^2 and three received 0.1mg/m^2 rituximab.

Second trial

In the randomized trial 75 healthy volunteers were screened and 36 were eligible for participation. Thirty-nine subjects were not randomized because the study arms matching the genetic polymorphism were closed for recruitment. The remaining 36 subjects were enrolled in the study. Three subjects had to be excluded from the per protocol analysis due to missing blood samples or unavailable CD19+/20+ cell counts. One of the three subjects voluntarily withdrew consent after completion of the trial day. All subjects had the V/F genotype and received the test product, one at 0.3 mg/m^2 and two at 0.1 mg/m^2.

Two subjects had to be excluded from the PK analysis due to erroneously obtained plasma instead of serum samples. Thus, data were available from 33 subjects in the PD analysis and of 31 subjects in the PK analysis.

Table S1. Demographics of participants

	First Trial (N = 16)	Second Trial (N = 36)
Gender m(f)	10 (6)	22 (14)
Age [years]	32 (20-49)	31 (20-49)
Height [cm]	176 (163-191)	175 (157-194)
Weight [kg]	74 (55-98)	70 (48-107)

Table S1. Baseline data of participants in both trials. Presented data are means (minimum - maximum).

Table S2. Subject disposition of the second trial

Description	Test Product N (%)	Reference Product N (%)
No. of Subjects Receiving medications	20 (56%)	16 (44%)
No. of Subjects Completing	19 (53%)	16 (44%)
Reasons for Discontinuation		
Discontinued for any reason	0 (0%)	0 (0%)
Withdrawal of Subject's Consent	1 (3%)	0 (0%)
Adverse Event	0 (0%)	0 (0%)
Major Non-compliance	0 (0%)	0 (0%)

Table S2. Subject Disposition after infusion of 0.1, 0.3 mg/m^2 of two rituximab products in 36 healthy volunteers.

Table S3. Mean CD20+ cell counts subdivided by the FCγRIIIa-158V/F polymorphism

Time	Polymorphism	Test-product		Reference product (Mabthera)	
		Mean cell counts	Percentage of baseline level	Mean cell counts	Percentage of baseline level
Baseline	combined	265	100%	213	100%
+1h	combined	131	50%	100	47%
Baseline	V/V	290	100%	284	100%
+1h	V/V	114	39%	107	38%
Baseline	V/F	224	100%	164	100%
+1h	V/F	117	52%	85	52%
Baseline	F/F	260	100%	190	100%
+1h	F/F	156	60%	108	57%

Table S3. Presented are absolute and relative (to the baseline) CD20+ cell counts subdivided by the FCγRIIIa-158V/F polymorphism.

Table S4. Changes in mean absolute CD20+ cell counts

	Proposed Biosimilar 0.1 mg/m^2 (n=10)	Reference Product 0.1 mg/m^2 (n=12)	Proposed Biosimilar 0.3 mg/m^2 (n=7)	Reference Product 0.3 mg/m^2 (n=4)
Baseline	265 (45.5%)	213 (60.7%)	237 (21.5%)	221 (26.7%)
End of infusion (+1 hour)	131 (31.8%)	100 (68.3%)	47 (40.8%)	28 (63.3%)
Four weeks after infusion	220 (37.6%)	162 (59.3%)	203 (24.9%)	188 (20.0%)

Table S4. Absolute CD20+ cell counts at the baseline, at the end of the infusion and four weeks after infusion of the test rituximab product or the reference product. Presented are means (coefficient of variation (CV) %).

Table S5. Pharmacokinetic parameters of test and reference product

Rituximab product		AUC_T [h*ng/mL]	AUC_{0-inf} [h*ng/mL]	C_{max} [ng/mL]	Cl [L/h]
Proposed Biosimilar $0.1 mg/m^2$ N=9	Mean±SD	323±575	1367±2084	26±8	0.0014±0.0018
	CV%	178	152	32	135
Reference Product $0.1 mg/m^2$ N=11	Mean±SD	213±574	357±799	32±11	0.0028±0.0015
	CV%	270	224	35	53
Proposed Biosimilar $0.3 mg/m^2$ N=7	Mean±SD	300±286	409±434	82±32	0.0029±0.002
	CV%	96	106	39	69
Reference Product $0.3 mg/m^2$ N=4	Mean±SD	193±19	273±52	89±6	0.0024±0.0005
	CV%	10	19	7	22

Table S5. PK of Test and Reference product at doses 0.1 mg/m^2 and 0.3 mg/m^2.

Table S6. Adverse Events of the first study

System organ class	Preferred term	Subjects (N=16)	Adverse Events (Total 56 AEs)
Blood and lymphatic system disorders	Lymphadenopathy	1 (6.3%)	1 (1.8%)
Gastrointestinal disorders	Nausea	3 (18.8%)	3 (5.4%)
General disorders and administration site conditions	Chills	1 (6.3%)	2 (3.6%)
	Fatigue	2 (12.5)	2 (3.6%)
	Influenza-like illness	1 (6.3%)	1 (1.8%)
	Vaccination site reaction left arm	1 (6.3%)	1 (1.8%)
Infections and infestations	Herpes Zoster	1 (6.3%)	1 (1.8%)
	Oral Herpes	1 (6.3%)	1 (1.8%)
Injury, poisoning and procedural complications	Contusion	1 (6.3%)	1 (1.8%)
Musculoskeletal and connective tissue disorders	Arthralgia	2 (12.5%)	2 (3.6%)
	Bursitis	1 (6.3%)	1 (1.8%)
	Cheekbone fracture	1 (6.3%)	1 (1.8%)
	Musculoskeletal chest pain	1 (6.3%)	1 (1.8%)
	Muskulosceletal pain	1 (6.3%)	1 (1.8%)
Nervous system disorders	Dizziness	2 (12.5%)	2 (3.6%)
	Headache	8 (50%)	24 (42.9%)
	Insomnia	1 (6.3%)	1 (1.8%)
	Migraine	1 (6.3%)	1 (1.8%)
Reproductive system and breast disorders	Dysmenorrhoea	1 (6.3%)	1 (1.8%)
Respiratory, thoracic and mediastinal disorders	Pharyngitis	3 (18.8)	3 (5.4%)
	Respiratory tract infection	3 (18.8%)	3 (5.4%)
	Rhinitis	1 (6.3%)	1 (1.8%)
Vascular disorders	Orthostatic hypotension	1 (6.3%)	1 (1.8%)

Table S6. Adverse events after infusion of 0.1, 0.3 or 1 mg/m^2 rituximab in 16 healthy volunteers.

Table S7. Adverse Events of the second trial

System Organ Class	Preferred Term	Subjects (N=32)	Adverse events (Total 50 AEs)
Gastrointestinal disorders	Diarrhea	1 (2.8%)	1 (2.0%)
	Dyspepsia	1 (2.8%)	1 (2.0%)
	Nausea	3 (8.3%)	3 (6.0%)
	Vomiting	1 (2.8%)	1 (2.0%)
General disorders and administration site conditions	Chills	1 (2.8%)	1 (2.0%)
	Fatigue	3 (8.3%)	3 (6.0%)
	Feeling Cold	1 (2.8%)	1 (2.0%)
	Feeling Hot	1 (2.8%)	1 (2.0%)
	Local swelling	1 (2.8%)	1 (2.0%)
	Pyrexia	1 (2.8%)	1 (2.0%)
Infections and Infestations	Abscess sweat gland	1 (2.8%)	1 (2.0%)
	Nasopharyngitis	2 (5.6%)	2 (4.0%)
	Urinary tract infection	1 (2.8%)	1 (2.0%)
Injury, poisoning and procedural complications	Muscle strain	1 (2.8%)	1 (2.0%)
Musculoskeletal and connective tissue disorders	Back pain	1 (2.8%)	1 (2.0%)
	Musculoskeletal pain	1 (2.8%)	1 (2.0%)
Nervous system disorders	Dizziness	1 (2.8%)	1 (2.0%)
	Headache	12 (33.3%)	18 (36%)
Reproductive system and breast disorders	Dysmenorrhoea	2 (5.6%)	2 (4.0%)
Respiratory, thoracic and mediastinal disorders	Asthma	1 (2.8%)	1 (2.0%)
	Cough	1 (2.8%)	1 (2.0%)
Skin and subcutaneous tissue disorders	Eczema	1 (2.8%)	1 (2.0%)
	Night Sweats	1 (2.8%)	1 (2.0%)
	Pruritus	1 (2.8%)	1 (2.0%)
	Rash	2 (5.6%)	2 (4.0%)
	Urticaria	1 (2.8%)	1 (2.0%)

Table S7. Adverse events after infusion of 0.1, 0.3 mg/m^2 of two rituximab products in 36 healthy volunteers.

Table S8. Safety Laboratory Parameters First Trial (N=16)

Time -point	CRP mg/dl	Hb g/dl	PLT $*10^9$/L	WBC $*10^9$/L	Lymphocytes $*10^9$/L	C3c mg/dl	C4 mg/dl	CH50 %
day 0 0h	0.08±0.24	14.0±1.3	240±56	6.1±2.0	2.0±0.7	105± 21	19±6	112
day 0 1h	n.d.	12.8±1.6	201±37	4.5±1.3	1.3±0.2	94± 20	17±6	103
day 0 2h	0.07±0.21	12.9±1.7	188±35	3.9±1.5	0.9±0.2	n.d.	n.d.	n.d.
day 1	1.59±1.55	14.1±1.6	226±44	4.1±1.2	1.5±0.3	111± 21	21±6	116
day 2	0.82±0.76	14.0±1.7	235±44	5.7±1.4	1.9±0.5	109± 20	21±6	116
day 3	0.47±0.42	14.1±1.6	238±43	5.7±1.4	2.1±0.6	110± 19	21±7	115

Table S8. Safety Laboratory parameters in the pilot trial (all groups pooled), infusion lasted for 1 hour starting at 0h. The table presents medians ± standard deviations. n.d.= not done, CRP= C-reactive protein, Hb=hemoglobin, PLT= platelet count, WBC= white blood cell count, C3c= Complement component (by nephelometry) 3, C4= Complement component 4 (by nephelometry), CH50%= total complement activity (by measuring the haemolytic activity in sensitized erythrocytes)

A pharmacokinetic model:

Assuming a circulating blood volume of 3000ml and a body surface area of 2 m^2 a dose of 1 mg/m^2 (2 mg rituximab in total) results in a theoretical maximum plasma concentration of 666 ng/ml. In our trial the measured C_{max} was lower (approx. 330 ng/ml mean, Figure 4), which may be explained by target dependent elimination. Moreover, a slowly declining plateau phase at a rituximab concentration of approximately 100 ng/ml (Figure 4) can be identified starting approximately six hours after the infusion. A concentration of 100 ng/ml in 3000 ml blood equals a total of 0.3 mg rituximab. Thus, after six hours 1.7 mg rituximab have been eliminated from the circulation. Moreover, at a concentration of 100 ng/ml CD20+ cells recovered, indicating that this concentration does not suffice to suppress B lymphocytes. However, at the C_{max} almost all CD20+ cells were depleted. Thus, doses close to the C_{max} should suffice to permanently suppress CD20+ cells.

When we increase the dose to 100 mg rituximab in the same person 100% of all CD20+ cells should be depleted. Moreover, one may expect a relatively lower target dependent elimination of rituximab from the circulation when higher doses are infused. Thus, the rituximab concentration of the plateau phase should increase more than ten-fold. Conservatively estimating that 8 mg rituximab are rapidly eliminated, a total of 12 mg rituximab would remain in the circulation. Assuming a plasma volume of 3000 mL a concentration of ~33μg/ml would result, which is one hundred-fold higher than the above-mentioned theoretical C_{max} of our trial. Taking the $T_{1/2}$ of rituximab of about 20 days into account, plasma rituximab concentrations should be maintained above 1000 ng/ml for 15 weeks. Based on our data, this concentration should suffice to deplete B-cells completely for at least 15 weeks and potentially even longer time periods.

Furthermore, if the next rituximab dose is given before CD20+ cells recover, target dependent elimination may be reduced and the concentration of the plateau phase should therefore be higher compared to the first dose. This may even prolong the dosing intervals further.

Figure 4 compares estimated plasma rituximab concentrations depending on different treatments. Plasma concentrations of the dosing regimen 375mg/m^2/week were adapted from Iacona et al.[3], 2x1000mg (day 1 and 15) from Cohen et al.[25], 2x100 mg were calculated based on the data presented in our trial and 1.0mg/m^2 were taken from our trial. A half-life of rituximab of 21 days was assumed.

Figure S1. Mean Plasma concentration-time curve

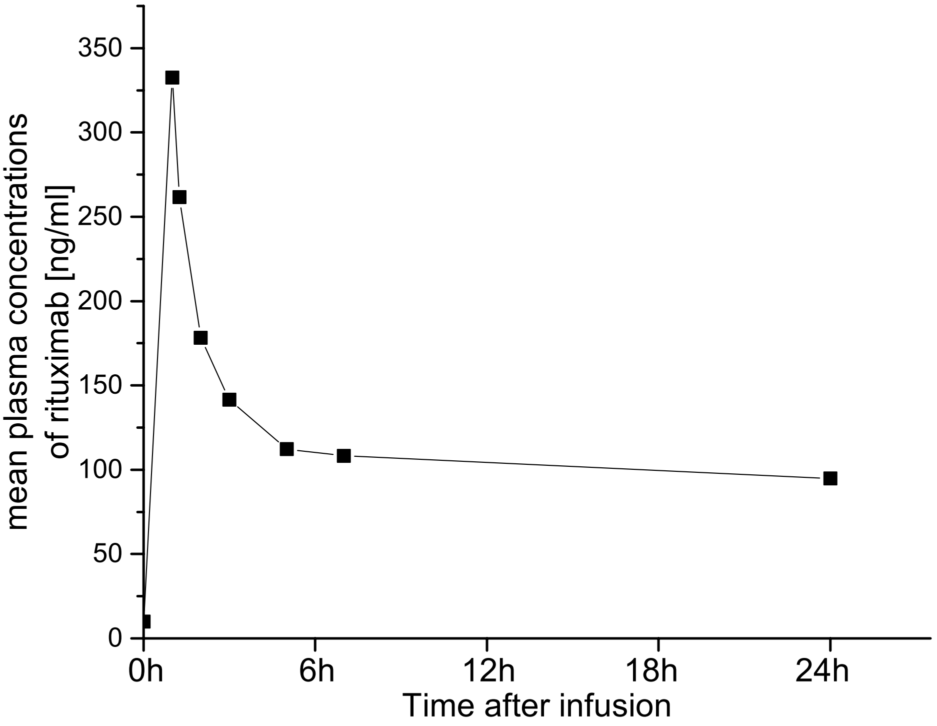

Figure S1. Plasma concentration-time curve after infusion of 1 mg/m^2 rituximab (n=8). Observations were censored after 24 hours because of a signal over noise ratio <5. Due to relative high analytical background of samples in rituximab naïve subjects, the analytical sensitivity of the used assay was insufficient to reliably determine PK parameters for the lower rituximab doses (0.1 and 0.3 mg/m^2).
